# CiteAb: a searchable antibody database that ranks antibodies by the number of times they have been cited

**DOI:** 10.1186/1471-2121-15-6

**Published:** 2014-02-14

**Authors:** Matthew A Helsby, Paul M Leader, Joe R Fenn, Tulay Gulsen, Chris Bryant, Gail Doughton, Ben Sharpe, Paul Whitley, Christopher J Caunt, Katrina James, Adam D Pope, Dave H Kelly, Andrew D Chalmers

**Affiliations:** 1 Department of Biology and Biochemistry, University of Bath, Bath BA2 7AY, UK; 2 CiteAb, Carpenter House, Bath BA1 1UD, UK; 3 Storm Consultancy, 14 New Bond Street, Bath BA1 1BE, UK

**Keywords:** Antibodies, Monoclonal, Polyclonal, Western blotting, Flow cytometry, Immunohistochemistry, ChIP host species, Species reactivity, Citations

## Abstract

**Background:**

Research antibodies are used by thousands of scientists working in diverse disciplines, but it is common to hear concerns about antibody quality. This means that researchers need to carefully choose the antibodies they use to avoid wasting time and money. A well accepted way of selecting a research antibody is to identify one which has been used previously, where the associated data has been peer-reviewed and the results published.

**Description:**

CiteAb is a searchable database which ranks antibodies by the number of times they have been cited. This allows researchers to easily find antibodies that have been used in peer-reviewed publications and the accompanying citations are listed, so users can check the data contained within the publications. This makes CiteAb a useful resource for identifying antibodies for experiments and also for finding information to demonstrate antibody validation. The database currently contains 1,400,000 antibodies which are from 90 suppliers, including 87 commercial companies and 3 academic resources. Associated with these antibodies are 140,000 publications which provide 306,000 antibody citations. In addition to searching, users can also browse through the antibodies and add their own publications to the CiteAb database.

**Conclusions:**

CiteAb provides a new way for researchers to find research antibodies that have been used successfully in peer-reviewed publications. It aims to assist these researchers and will hopefully help promote progress in many areas of life science research.

## Background

Research antibodies are used by life scientists who work in areas ranging from cell biology to immunology and from neuroscience to cancer research. The sheer scale of their use is illustrated by the fact that the market for commercial research antibodies is estimated to be over $1.6 billion annually [1]. However, there are often complaints from researchers about antibody quality [2-4] and new researchers learn that it is important to find well validated antibodies or risk wasting money and perhaps more importantly, time [5]. The selection of an antibody is complicated by the fact that they are used for a wide variety of applications, including western blotting, flow cytometry, ELISA, immunoprecipitation, chromatin immunoprecipitation (ChIP) and immunohistochemistry [6-9] and suitability for one application is not a guarantee of good performance with another application. In addition, antibodies are often used against an antigen from a different species to the one that the antibody was raised against: meaning that the amount of cross species reactivity shown by the antibody needs to be considered. Many researchers would agree that the most reliable way of overcoming these issues and identifying a suitable antibody would be to find one that has already been used for the application/species required and the results published in one or more publications.

CiteAb is a searchable antibody database that ranks antibodies by the number of times they have been cited in peer-reviewed journal articles, making it easy to find antibodies that have been successfully used. This approach to finding antibodies has advantages over a Google search, which is dependent on many factors which do not directly relate to antibody quality. For example, the supplier with the best search engine optimisation does not necessarily provide the most suitable antibody. There are existing specialist databases which rank antibodies based on commercial criteria and there are also others which help scientists find independently validated antibodies by collecting user reviews. CiteAb takes an alternative approach and focuses entirely on using peer-reviewed publications as a guide to the level of independent validation. Ranking by citations provides a simple and transparent method of helping researchers find an antibody that has been independently validated for a particular experimental method and/or species of interest. A citation means that the antibody has generated data worthy of publication in a peer-reviewed journal, which in our opinion provides the best guide when selecting an antibody. Having identified a potential antibody of interest, researchers can then link to the listed scientific papers and establish how the antibody has been used and examine the data contained within the publications. In addition to search, the key features of CiteAb include the ability to browse for antibodies of interest and for users to improve the database by adding information on their own publications if they are not already included. This article will provide an overview of the data currently stored in the CiteAb database, the different ways users can interact with it and finally a comparison of the results obtained from a range of different antibody search engines.

## Construction and content

CiteAb has a simple database architecture consisting of an entry with associated data fields for each antibody and publication. The antibody pages are searchable and linked to the relevant publication pages (the user workflow is described below). To maximise CiteAb’s utility for researchers as many antibodies as possible are listed and antibodies from any commercial company or academic resource can be included. Current statistics (December 2013) show that CiteAb contains 1,400,000 antibodies from 90 suppliers. These suppliers include 87 commercial companies and 3 academic resources. The academic resources are normally grant funded or non-profit self-funded centres and currently include the Developmental Studies Hybridoma Bank (DSHB) based at the University of Iowa, NeuroMab based at the University of California Davis and the Zebrafish International Resource Centre (ZIRC) from the University of Oregon, which works closely with the Zebrafish Model Organism Database (ZFIN) [10]. These resources are all currently funded, or were funded, by the National Institute of Health (NIH). For example the DSHB was funded by the NIH, but is now self-funded. More recently we have tried to encourage individual academics to list their antibodies and created a special category ‘academic antibodies (individual labs)’ to facilitate this. For this type of listing we ask that the academic be willing to distribute their antibody on request.

The second data type required to maximise the usefulness of CiteAb is to link as many publications as possible to these antibodies. CiteAb currently (December 2013) contains 140,000 publications, which provide 306,000 antibody citations. Despite the importance of citations, CiteAb does list antibodies which currently lack them. This is because newly released antibodies will not have citations, but are still likely to be of interest to users. In addition having these antibodies listed makes it easier for users to add citation data to them.

The data in CiteAb is obtained from multiple sources including the suppliers, academic resources, from users adding information on the antibodies they have used (discussed below) and from our curation of antibodies and citations. Different approaches to data collection are used as no single method can identify all the publications that use antibodies, and establish the application and species used within the publications. An example of using a particular data source is the information kindly provided by Xenbase [11], whose data lists publications where antibodies were used with the model organism Xenopus. Quality control for the information obtained from the different data sources is ensured using two complementary mechanisms. Data is batch tested to ensure it is reliable and prominent “report a problem” buttons are displayed on the antibody pages so users can report any errors that do make it into the database. The ability to check the source data is a key advantage of using citations as a guide to the likely suitability of an antibody. The amount of data contained within CiteAb has grown rapidly since the launch of the trial site in September 2012 (Table 1 and our statistics page (blog.citeab.com/citeab-database-statistics) and our most important future goal is to continue to increase the number of antibodies and publications which are contained within the database.

**Table 1:** Growth of the CiteAb database

**Development stage**	**Date**	**Number of antibodies**	**Number of companies**	**Number of academic resources**	**Number of individual academic labs**	**Number of publications**	**Number of antibody citations**
**Trial site launched**	1/9/12	400,000	35	1	-	35,000	-
**Version 1 launched**	11/3/13	900,000	51	3	-	115,000	237,000
**Addition of extra antibody information fields**	5/8/13	1,125,000	72	3	1	123,000	260,000
**Resubmission of the CiteAb paper**	11/12/13	1,400,000	87	3	1	140,000	306,000

The table shows how the amount of antibody and citation data that is included in CiteAb has increased over time. Current information can be viewed on our statistics page (blog.citeab.com/citeab-database-statistics).

## Utility and discussion

### Searching CiteAb for antibodies

**Figure 1:**
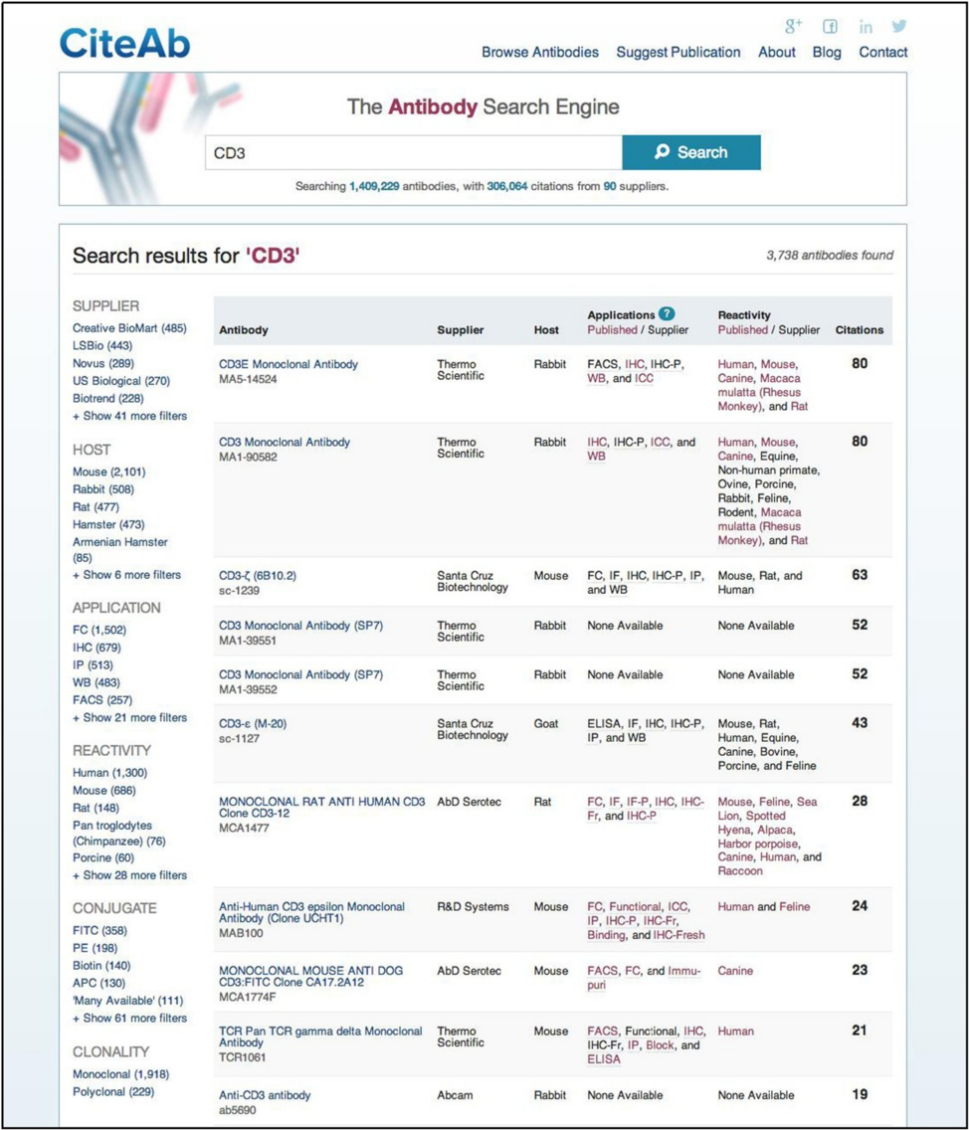
**A CiteAb search results page.** The antibodies returned by a search are displayed in a table and ranked by the number of times they have been cited (far right column). The following antibody information is displayed; the antibody name (blue text), antibody code (below the name), name of the supplier, host species and information on applications and species reactivity. Application and reactivity data includes supplier recommendations (black text) and data from peer-reviewed publications (red text). Filters (far left hand side) are available for the supplier, host species, application, species reactivity, conjugate and clonality.

The main way for users of CiteAb to access information is via the search function. This feature can be accessed from any page and requires researchers to input a search term in the prominent search box (http://www.citeab.com/). Fields for the antibody name, supplier code, clone number, synonym and immunogen for each antibody within the CiteAb database are then searched for the entered term. The antibodies returned by a search are ranked by the number of times they have been cited and are presented in a table which displays key antibody information (Figure 1). This information includes; the antibody name (blue text), antibody code (under antibody name), name of the supplier who provides the antibody, host species, applications, species reactivity and number of citations. The information on applications and species reactivity includes data from peer-reviewed publications (red text) and recommendations provided by the supplier (black text). The search results can be filtered by the following features; the supplier, host species, experimental application, species reactivity, conjugate and clonality.

**Figure 2:**
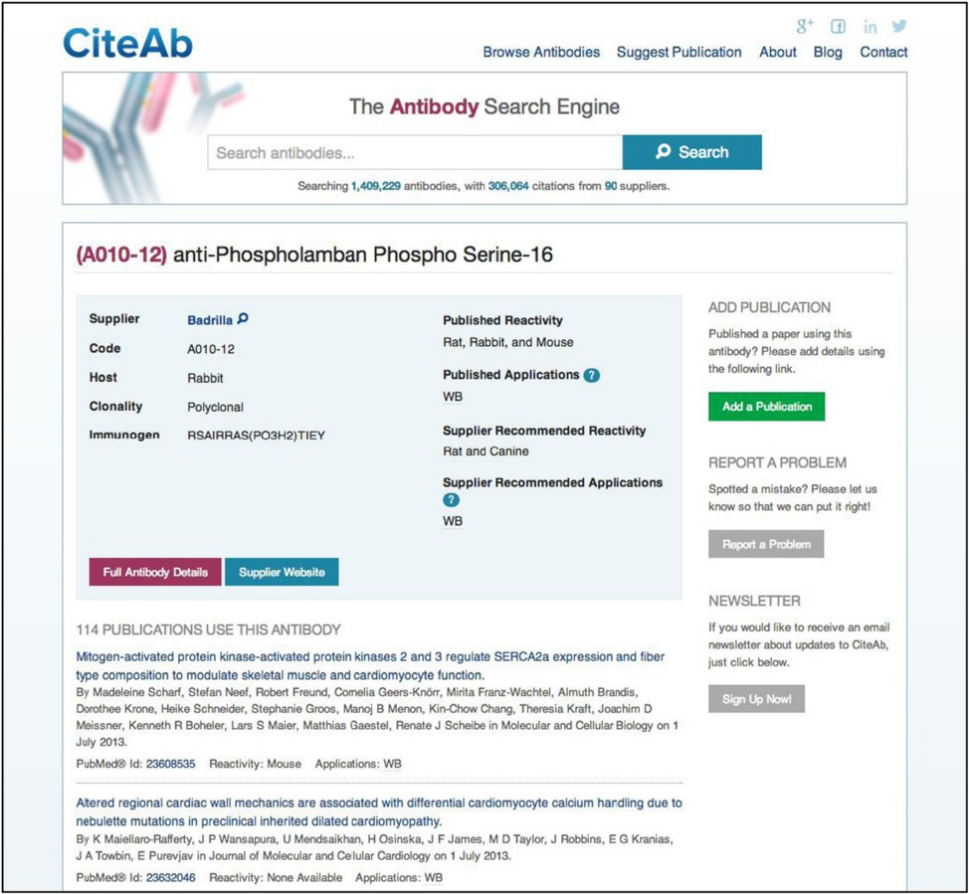
**Example of a CiteAb antibody page.** Each antibody page provides details of the antibody code, antibody name, name of the supplier, host species, whether the antibody is polyclonal or monoclonal, clone number if monoclonal (not present in this example), any conjugated moiety such as FITC (not present in this example), synonyms for the antigen (not present in this example) and information on the immunogen. Published reactivity, published applications, supplier recommended reactivity and supplier recommended applications are also provided where available. Not all antibodies will have complete information as several of these data fields have been added recently and uploading of the additional information is continuing. The antibody page also lists publications that have cited the antibody. The first ten are shown automatically, with an option to view the remainder. Each publication has a link to a corresponding ‘publication page’ (Figure 3). There are also links to add a publication, report a problem and sign up to the CiteAb newsletter.

Having identified an antibody of interest, users are then able to navigate to the ‘antibody page’ (Figure 2) by clicking on the relevant row for the antibody. An antibody page displays the antibody code, antibody name, name of the supplier, host species, published applications and published species reactivity. A recent upgrade of the database has added fields for the following; whether the antibody is polyclonal or monoclonal, clone number if monoclonal, any conjugated moiety such as Fluorescein isothiocyanate (FITC), synonyms for the target protein and information on the immunogen. These fields are only displayed if the information is present in the database, for the example shown in Figure 2 fields for synonym, clone number and conjugated moiety are not displayed. Fields for supplier recommended applications and supplier recommended species reactivity have also recently been added. Uploading of information for the new fields is ongoing, so not all antibodies will have this additional information. If the antibody appears worthy of further investigation the user can follow links to the corresponding antibody page on the supplier website for further information.

**Figure 3:**
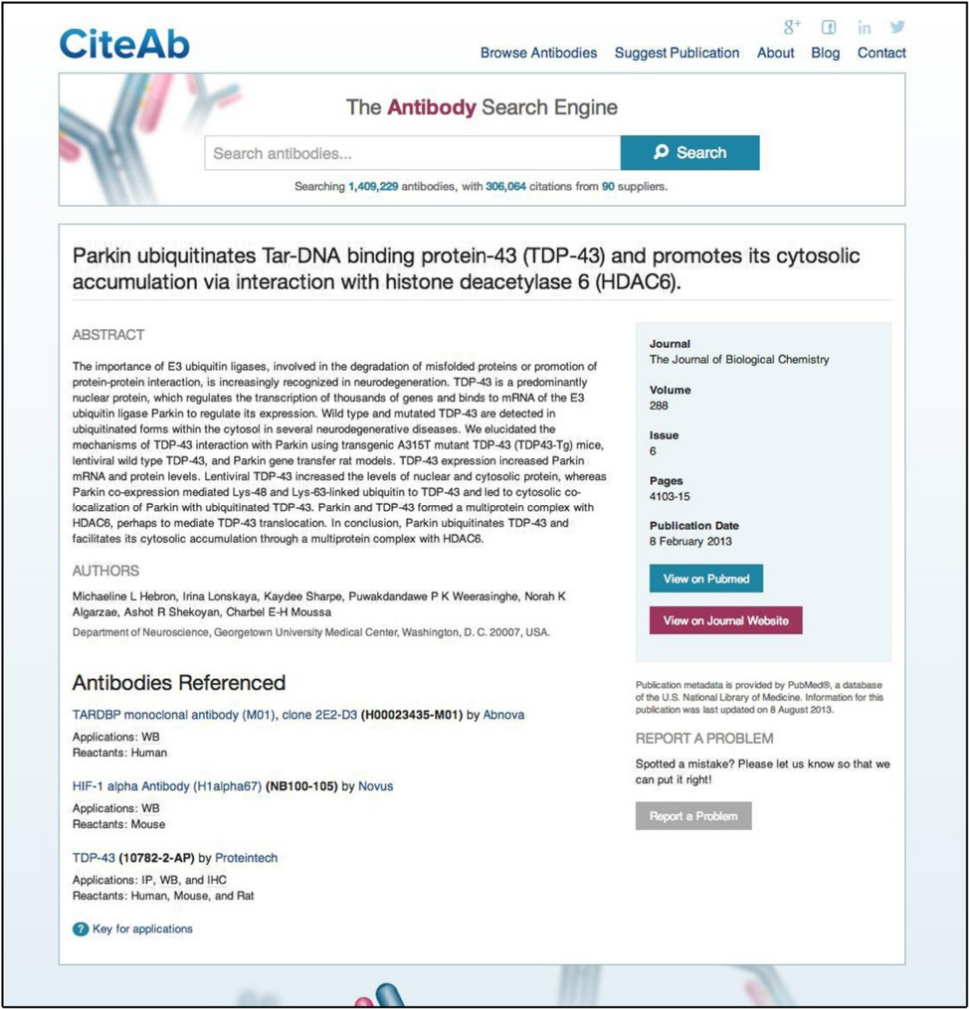
**A CiteAb publication page.** The publication pages display the article title, abstract, authors, journal and a list of the antibodies cited in the publication. This page also includes links to the relevant entry in PubMed and to the publishing journal’s article page. The publication metadata is provided under license by PubMed [12].

The antibody pages have a ‘Report a Problem’ button, enabling users to highlight any errors in the information displayed and an ‘Add a Publication’ button which allows users to add information on their own publications if they are not already contained within CiteAb (see below). They also list the publications that have cited the antibody and each publication has a link to a corresponding ‘publication page’. The publication pages (Figure 3) give information on each publication, including article title, abstract, authors, journal and a list of the antibodies cited in the publication. This page also includes links to the relevant entry in PubMed and to the publishing journal’s page for the article. The publication metadata is provided under licence by PubMed [12] a database of the U.S. National Library of Medicine.

### Browsing antibodies in CiteAb

**Figure 4:**
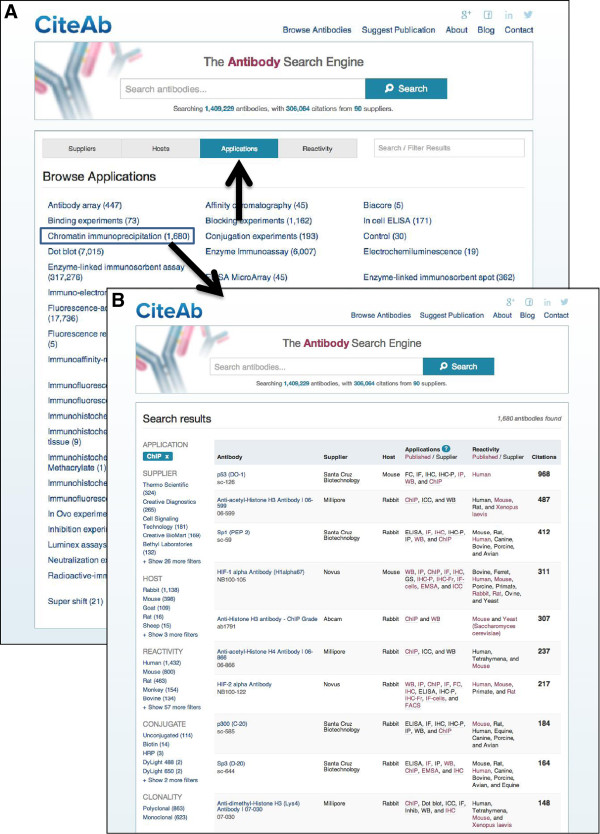
**Browsing antibodies in CiteAb. (A)** Selecting a feature to browse by, for example ‘applications’ (upward arrow), returns an alphabetical list of available applications. **(B)** Selecting a specific application, such as ‘Chromatin Immunoprecipitation’ (blue box), then returns a list of antibodies that may be suitable for this application. This data includes applications recommended by the suppliers and applications from publications. Users can add further filters and search terms as required.

The second way in which users are able to access information in CiteAb is via a browse function (http://www.citeab.com/browse/companies). This allows users to browse for antibodies of interest via the supplier, the host species, the application or the species reactivity. Having selected a feature to browse by, for example ‘applications’, an alphabetical list of available applications is displayed (Figure 4A). Selecting a specific application, such as ChIP, then returns a list of antibodies that are potentially suitable for this application (Figure 4B). The user is then able to browse through these antibodies or add additional filters or search terms, to identify specific antibodies of interest.

The browsing options do not include an alphabetical list of antibodies because antibody names do not follow a consistent format. For example, a polyclonal goat anti-p53 antibody might be named anti-p53 polyclonal antibody, goat anti-p53 polyclonal antibody, p53 polyclonal antibody or polyclonal anti-p53 antibody. This variation, combined with the large numbers of antibodies in many categories, makes alphabetical browsing potentially misleading and in our opinion of little value.

### Users can add their publications to CiteAb

**Figure 5:**
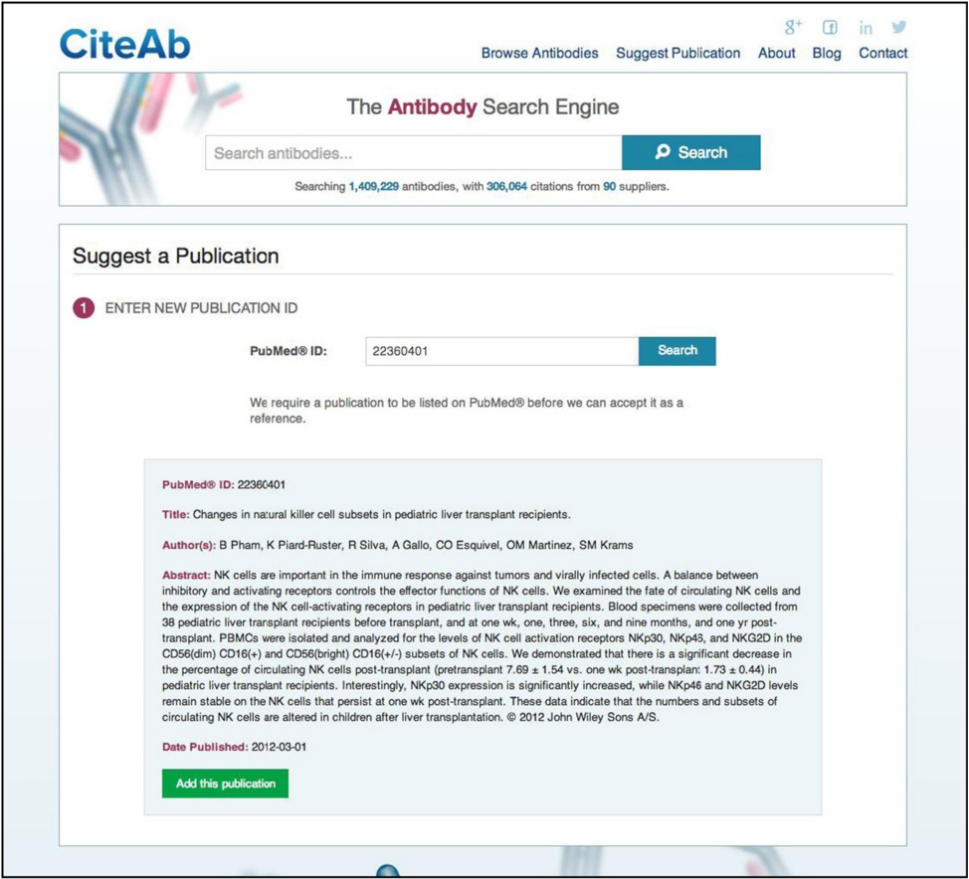
**Adding publication information to CiteAb (Step 1).** A three step submission process is used to enter publication information. In step one a user enters the PubMed ID of a publication and confirms it is correctly identified. Figure 6 illustrates steps two and three.

**Figure 6:**
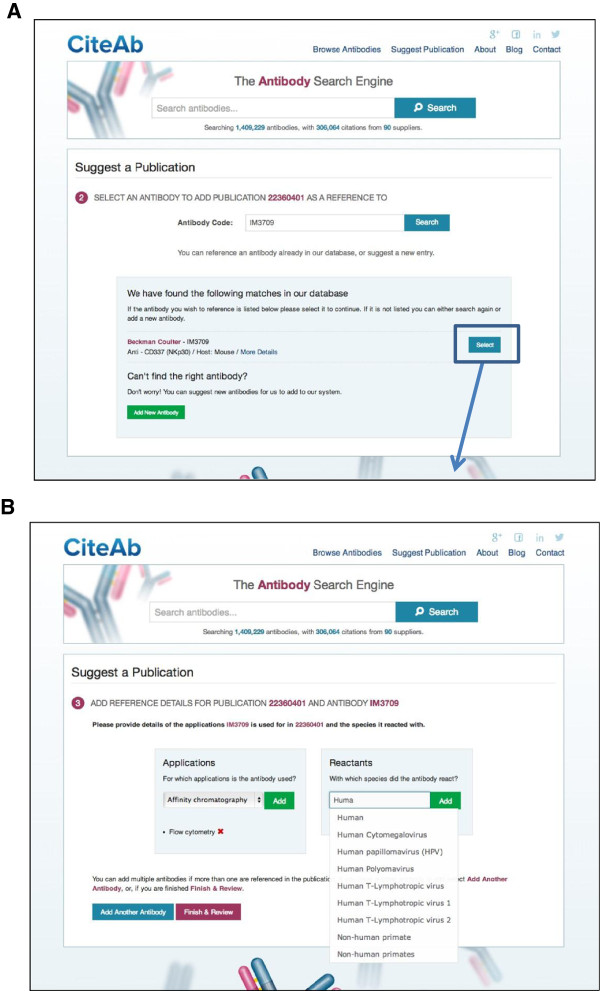
**Adding publication information to CiteAb (Steps 2 + 3).** A three step submission process is used to enter publication information. **(A)** In step two an antibody used in the publication is selected by entering its code number and selecting the relevant antibody. **(B)** In step three, information on the application and species reactivity for the antibody is added. Steps two and three are repeated for each antibody used in the publication and the information is then reviewed and submitted. Figure 5 illustrates step one of the submission process.

A third way in which users can interact with CiteAb is to add information on the antibodies used in their publications if they are not already contained within the database (http://www.citeab.com/suggest_publication). This is carried out using a three step submission process. First, the user enters the PubMed ID of their paper and confirms it is correctly identified (Figure 5). They then select an antibody used in the publication by its code number (Figure 6A) and information on the application and species reactivity for the antibody can be added (Figure 6B). The second two steps are repeated for each antibody used in the publication, before the information is reviewed and submitted. The process was designed so that users do not have to enter detailed publication or antibody information in order to make the process as quick and simple as possible and to hopefully reduce data entry errors.

If an antibody used in the publication is not currently listed in CiteAb, then a new antibody can be added to the database. In this case the user is asked for key information regarding the antibody including the supplier of the antibody, antibody name, antibody code number and host species. This allows researchers to add any antibody they have used, including those they have raised in their own laboratories.

We try and encourage our users to add information on their publications as the more researchers that add citations to CiteAb the greater its use will be to the scientific community. Users who add their citations may also benefit as their work can then be accessed by other researchers who are using the site, potentially increasing the impact of their publications.

### Other features

The ‘About Us’ section (http://www.citeab.com/about) provides users with information regarding how the CiteAb antibody search engine functions, the people who work on the CiteAb database and how to list antibodies. It also has links to a help page, a database statistics page, an acknowledgement page and a page providing further advice on listing antibodies. There is a prominent ‘Contact’ page (http://www.citeab.com/contact) enabling users to ask for additional information if required. CiteAb also has a Blog (blog.citeab.com/) and a newsletter (http://www.citeab.com/newsletter) which are designed to provide information on CiteAb database developments, antibody supplier news and journal/publishing features that may be of interest to users.

### A useful source of information regarding antibody validation

CiteAb is designed to help researchers find antibodies for their research. However, it has another use which is as a source of information regarding antibody validation. The Nature Publishing Group have recently introduced a reporting checklist for articles [13], which highlights the fact that reporting of research antibodies in publications needs to be improved and we have subsequently suggested a standardised format [14]. One of the recommendations from the checklist, and our article, is that authors need to show that the antibodies they use have been properly validated. Antibody validation is a complicated topic [15-19], but one way of demonstrating validation is to cite papers in which the antibody has been previously validated. Another is to cite the antibody page from a database that provides evidence of past use. In both these cases CiteAb can help researchers show antibody validation.

### Comparison with the results from other antibody search engines

The task of selecting an antibody is far from straightforward and, as mentioned above, several existing antibody search engines have been developed to help this process. The way search engines rank antibodies falls into three rough categories. Some use a commercial model where suppliers pay to be top ranked while others use user reviews to provide independent validation and a method of ranking. In contrast CiteAb uses citations for independent validation and ranking. A search engine called BioBrea also uses a similar approach and ranks by citations. Search engines may also mix different models by allowing suppliers to pay to be top ranked/highlighted, but rank the remainder of the antibodies by reviews or citations. The different approaches have different advantages and disadvantages. User reviews can provide valuable independent information regarding validation of the antibody, but they can be hard to collect and it may be difficult to assess the validity of the review. Citations are independent and Citeab displays them so users can check the data contained within the papers. However, citations may take time to appear for new antibodies. The paid models allow companies to promote new antibodies which will initially lack citations and reviews but may be the best available product.

To allow a comparison of the results obtained from different antibody search engines 14 of them were tested with three search terms (Table 2). Two search terms are for commonly used antibodies (1, search CD4 and filter for flow cytometry. 2, search GAPDH and filter for Western blotting) and the third was less widely used (search STMN3). The striking thing about the results is the variety of antibodies that were returned as the top hit by the different search engines (Table 2). There is almost no overlap in the results which are returned. It is not possible to say which is the “best” antibody search engine from this analysis. The results from each search would need to be experimentally tested in an independent laboratory. However, it does demonstrate how challenging it is to select an antibody to buy. Our opinion is it is advisable for researchers to use multiple search engines, look into the data behind the results and consider several antibodies if available. This allows an informed decision to be made. It is also important to stress that once purchased the antibody should still be validated for the application and species being used within the purchasing lab. This information, along with validation of new antibodies, can then be included as part of future publications to help other researchers.

**Table 2:** Search results from a range of antibody search engines

**Antibody search engine**	**CD4 for flow cytometry**	**GAPDH for Western blotting**	**STMN3**
1DegreeBio (http://www.1degreebio.org)	BioLegend (100405)	Abcam (ab9485)	Atlas Antibodies (HPA012947**)**
Antibody Directory (http://www.antibodydirectory.com)	Cell Sciences (MON2067)	LabFrontier Life Science Institute (LF-PA0006)	None
Antibody Registry (http://www.antibodyregistry.org)	GeneTex (GTX21089)	MBL International (JM-3777-100)	LifeSpan BioSciences (LS-C39280-50)
Antibody Resource (http://www.antibodyresource.com)	SICGEN (AB0091-200)	SICGEN (AB0067-200)	Abbexa (abx14926)
Antibody Review (http://www.antibodyreview.com)	LifeSpan Biosciences (LS-C21574)	Creative Biomart (CPBT-53909RH)	None
Antibodypedia (http://www.antibodypedia.com)	Novus Biologicals (NBP1-19371)	Proteintech (10494-1-AP	Atlas Antibodies (HPA012947)
Benchwise (http://www.benchwise.org)	BD Biosciences (552775)	Abcam (ab8245)	None
BioBrea (http://www.biobrea.com)	BD Biosciences (550280)	Abcam (ab8245)	Proteintech (11311-1-AP)
Biocompare (http://www.biocompare.com)	Antibodies-online (ABIN641732)	Antibodies-online (ABIN569034)	Biorbyt (orb31054)
**CiteAb (**http://http://www.citeab.com **)**	**AbD Serotec (MCA1749)**	**Santa Cruz Biotechnology (SC-25778)**	**Proteintech (11311-1-AP)**
Labome (http://www.labome.com)	LifeSpan Biosciences (LS-B3426)	Abbiotec (252626)	Santa Cruz Biotechnology (SC-85907)
Linscott Directory (http://www.linscottsdirectory.com)	BACHEM AMERICAS (T-1364.0100)	Synaptic Systems (247 002)	Proteintech (11311-1-AP)
pAbmAbs (http://www. pabmabs.com)	None	Sigma (G8795)	None
Scrazzl (http://www.scrazzl.com)	Santa Cruz Biotechnology (sc-19642)	Abcam (ab83957)	Abcam (ab76678)

Three searches were carried out with a range of antibody search engines and the top ranked antibody recorded. The first two searches were for commonly used antibodies (CD4/filter flow cytometry) and (GPDH/filter Western blotting). The third is a less common search (STMN3) which was randomly selected (it was the 100^th^ most common search in CiteAb in the preceding three months). Results from CiteAb are highlighted in bold. Searches were carried out in December 2013.

### Future directions

Our major future goal is to continue to improve the quantity and quality of the antibody and citation data contained within the CiteAb database. In terms of technical improvements, we want to improve the ability of users to browse and filter publications that are associated with an antibody. In the longer term the database is also likely to adapt to accommodate changes in available antibodies, such as increasing numbers of recombinant antibodies and also adding other types of affinity reagents. There is no doubt that there are other improvements that could be made to CiteAb and we would appreciate any suggestions from readers of this article.

## Conclusions

The goal of CiteAb is to help researchers succeed by making it easier for them to find the right research antibody for their experiments. It does this by ranking research antibodies by the number of times they have been cited and making it easy for researchers to explore the citations. This database will hopefully save scientists time and money and by doing this help life science research progress more rapidly. Our initial feedback has been that researchers find CiteAb a useful resource and our analytics data shows that we have increasing numbers of repeat visitors. Our aim is to continue to improve CiteAb and make it as valuable to researchers as possible.

## Availability and requirements

The CiteAb database can be accessed at http://www.citeab.com. It is freely available for use by academics and non-academics without the need for login or registration.

## Abbreviations

ChIP: Chromatin immunoprecipitation; DSHB: Developmental Studies Hybridoma Bank; FITC: Fluorescein isothiocyanate; NIH: National Institute of Health; ZFIN: Zebrafish Model Organism Database; ZIRC: Zebrafish International Resource Centre.

## Competing interests

CiteAb is in the process of being spun out from the University of Bath. Following spinout ADP, DHK and ADC will be shareholders in the newly formed company.

## Authors’ contributions

MAH: managed and carried out upload of data, contributed ideas to database design and function and helped draft the manuscript. PML: contributed ideas to database design and function, and wrote code for the database. JRF: assisted in data uploading and helped draft the manuscript. TG: contributed to data uploading. CB: contributed to data uploading. GD: contributed to data uploading. BS: contributed to data uploading. PW: contributed ideas to database design and function. CJC: contributed ideas to database design and function. KJ: contributed ideas to database design and function and helped draft the manuscript. ADP: contributed ideas to database design and function, and wrote code for the database. DHK: contributed ideas to database design and function, and coordinated the project. ADC: conceived the idea for the database, contributed to design and function of the database and drafted the manuscript. All authors read and approved the final manuscript.

## Authors’ information

MAH: immunology graduate. PML: computer scientist. JRF: molecular cell biology graduate. TG: cell biology PhD student. CB: cell biology PhD student. GD: cell biology PhD student. BS: cell biology PhD student. PW: senior lecture in cell biology. CJC: lecturer in cell biology. KJ: digital communications expert. ADP: computer scientist. DHK: digital technology expert. ADC: lecturer in cell and developmental biology.
